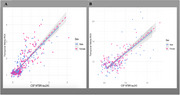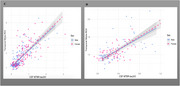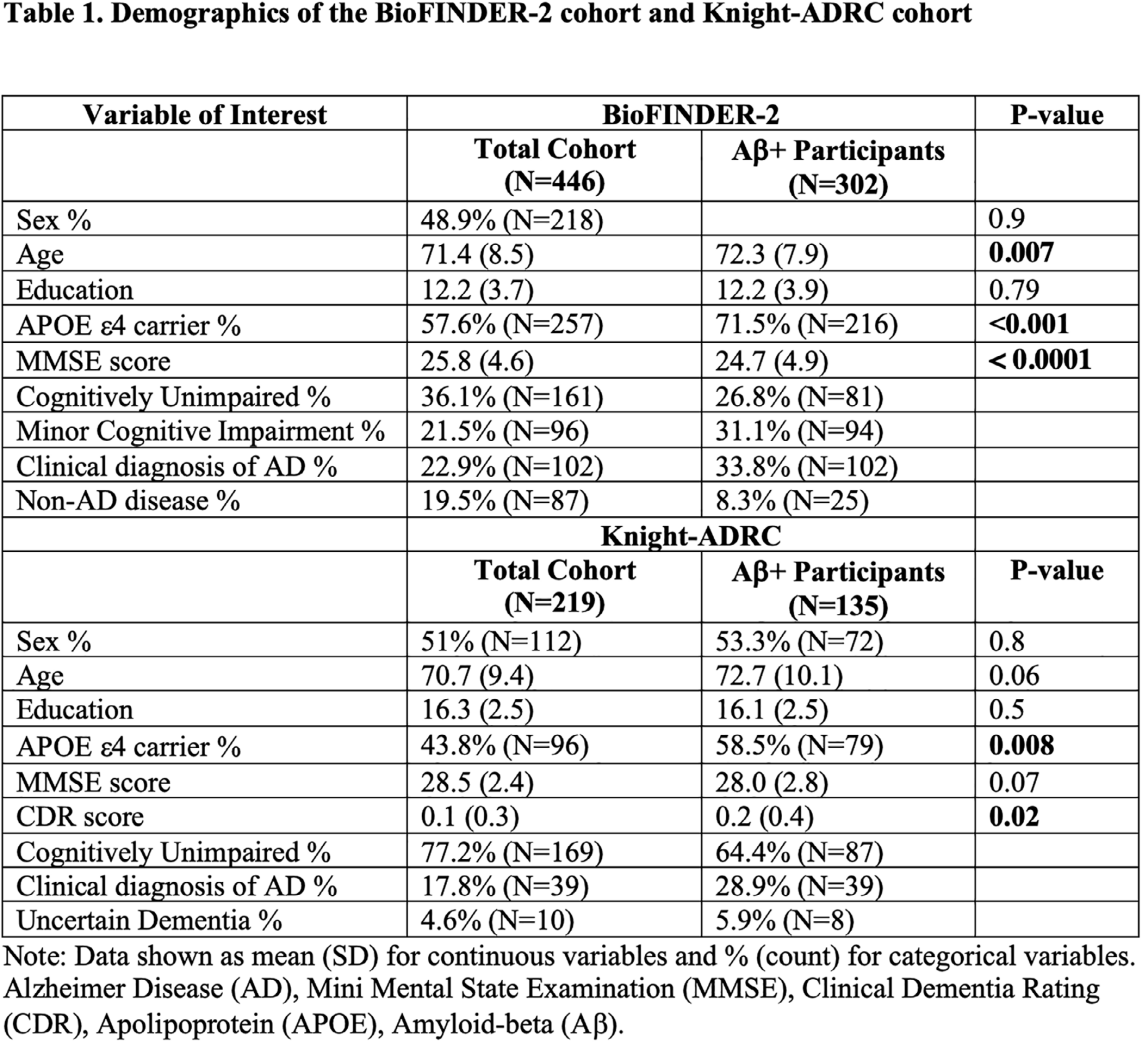# Associations between tau PET and CSF MTBR243 do not vary by sex

**DOI:** 10.1002/alz70856_098213

**Published:** 2025-12-24

**Authors:** Carling G Robinson, Alexa Pichet Binette, Kanta Horie, Chihiro Sato, Suzanne E. Schindler, Nicolas R. Barthélemy, Randall J. Bateman, Tammie L.S. Benzinger, Shorena Janelidze, Ellen Singleton, Erik Stomrud, Sebastian Palmqvist, Niklas Mattsson‐Carlgren, Oskar Hansson, Brian A. Gordon, Rik Ossenkoppele

**Affiliations:** ^1^ Washington University in St. Louis School of Medicine, St. Louis, MO, USA; ^2^ Université de Montréal, Montréal, QC, Canada; ^3^ Clinical Memory Research Unit, Department of Clinical Sciences Malmö, Faculty of Medicine, Lund University, Lund, Sweden; ^4^ Centre de Recherche de l’Institut Universitaire de Gériatrie de Montréal, Montréal, QC, Canada; ^5^ Eisai Inc., Nutley, NJ, USA; ^6^ The Tracy Family SILQ Center, St. Louis, MO, USA; ^7^ Washington University in Saint Louis, Saint Louis, MO, USA; ^8^ Department of Neurology, Washington University School of Medicine, St. Louis, MO, USA; ^9^ Washington University in St. Louis, St. Louis, MO, USA; ^10^ Department of Radiology, Washington University School of Medicine, St. Louis, MO, USA; ^11^ Memory Clinic, Skåne University Hospital, Malmö, Sweden; ^12^ Clinical Memory Research Unit, Department of Clinical Sciences Malmö, Lund University, Lund, Lund, Sweden; ^13^ Memory Clinic, Skåne University Hospital, Malmö, Skåne, Sweden; ^14^ Clinical Memory Research Unit, Department of Clinical Sciences Malmö, Faculty of Medicine, Lund University, Sweden, Lund, Sweden; ^15^ Clinical Memory Research Unit, Lund University, Malmö, Skåne, Sweden; ^16^ Department of Radiology, Washington University in St. Louis, St. Louis, MO, USA; ^17^ Amsterdam Neuroscience, Neurodegeneration., Amsterdam, Netherlands; ^18^ Alzheimer Center Amsterdam, Neurology, Vrije Universiteit Amsterdam, Amsterdam UMC location VUmc, Amsterdam, Netherlands

## Abstract

**Background:**

Prior research, replicated across multiple cohorts, has shown sex differences in tau Positron Emission Tomography (PET), with females exhibiting greater tau tracer binding in medial temporal and neocortical regions, particularly in those with positive amyloid‐beta (Aβ) biomarkers. Although typically interpreted as a potentiation of Alzheimer disease (AD) pathology, it remains unclear whether PET methodological factors or underlying biological mechanisms contribute to these observed sex differences. Microtubule binding region tau species containing residue 243 (MTBR‐tau243) in cerebrospinal fluid (CSF) is a biomarker of AD tau pathology and has been shown to be associated with tau‐PET. However, the potential relationship between MTBR‐tau243 and the observed sex differences in tau‐PET, remain unexplored. To address this gap, we conducted a cross‐sectional analysis of CSF MTBR‐tau243 and tau‐PET by sex in two cohorts: participants from the Swedish BioFINDER‐2 Study and Charles F. and Joanne Knight Alzheimer Disease Research Center (Knight‐ADRC).

**Method:**

Participants were required to have baseline data for both CSF MTBR‐tau243 and tau‐PET, as well as Aβ status defined by the CSF Aβ42/40 ratio. Tau‐PET was measured using the Flortaucipir tracer in the Knight‐ADRC cohort and the RO948 tracer in the BioFINDER‐2 cohort. In both cohorts, clinical diagnoses were reviewed to identify cases of AD dementia or other dementias. The main analysis of interest was whether there was a significant interaction between sex and CSF MTBR‐tau243 in predicting a temporal meta‐ROI, with additional analyses examining this interaction in Aβ‐positive participants.

**Result:**

In both cohorts there were significant associations between CSF MTBR‐tau243 and the temporal meta‐ROI (BioFINDER‐2: t = 17.1, *p* < 0.001, Knight‐ADRC: t=11.0, *p* <0.001). This association was not different by sex (BioFINDER‐2: t = 0.54, *p* =  0.6, Knight‐ADRC: t=0.1, *p* = 0.9) (Figure 1). This finding remained consistent within the Aβ‐positive subsets of both cohorts (Figure 2).

**Conclusion:**

Across two cohorts we found that sex did not moderate the relationship between tau‐PET and CSF MTBR‐tau243, two distinct markers of aggregated tau pathology. This suggests the sex effects found in tau‐PET likely represent a true biological finding not specifically tied to tau‐PET methodology.